# Surgical Fracture of Femur

**Published:** 1885-07

**Authors:** R. M. Swearingen

**Affiliations:** State Health Officer, Austin, Texas


MEDICAL TEXAS JOURNAL.

*EDITED AND PUBLISHED MONTHLY BY*

F. E. DANIEL, M. D., : AUSTIN, TEXAS.

Vol. I.] JULY, 1885. [No. I.

"*Scribimus indocti, doctique!*"

### Original Articles.

Contributed Exclusively to this Journal.

*The Articles in this Department are accepted, and published with the understanding that we are not responsible for, nor do we indorse the views and opinions of the writers, by so doing.*

### SURGICAL FRACTURE OF FEMUR.

*A Case: by R. M. Swearingen, M. D.,*

STATE HEALTH OFFICER, AUSTIN, TEXAS.

Read before the Travis County Medical and Surgical Society.—Reported for Daniel's Texas Medical Journal.

One year ago I was called to a young lady, twelve years of age, Miss L., who had sustained a comminuted fracture of the upper third of the femur. The injury was caused by a heavy heating register weighing nearly a thousand pounds falling upon her; the sharp-edged crown, not only crushing the bone, but almost severing the muscles.

The contusion of the parts, in my judgment, made it unsafe to use the plaster dressing, and the long splint, from the axilla to the foot, was applied.

The patient had no ugly symptoms or complications, and on the fortieth day the splint was removed.

Adhesions were firm, and accurate measurement revealed only one inch shortening.

I instructed the young lady to remain on the bed one week longer, and make no endeavor to walk, or even to bear the weight of the body upon the broken limb.

These extraordinary precautions were taken because of an apprehension, on my part, that the coaptation (owing to the oblique character of fracture) was incomplete.

Three weeks afterwards, hearing that the young lady was unable to walk without a crutch, I again called and examined her. To my astonishment and humiliation, the leg was fully three inches short; everted almost to a right angle, with strong union.

A series of cross questions elicited the confession on her part that the night after the removal of the splint, in trying to walk, she had fallen and broken the old adhesions.

She remained in bed sufficiently long for new callus, from the second fracture, to make the abnormal connections above described.

I called Dr. McLaughlin, and after consultation, the Doctor gave chloroform, and for the third time within a period of sixty days the femur was again broken.

I made extension, and fixed it, so that retraction was impossible, until the plaster dressing from the foot to the waist, had time to harden. Complete immobility was thus secured, and the desired extension made permanent. Thirty days afterward, we again removed the bandages, and, to our great gratification, found a perfect success, as to union; and the two limbs precisely the same length.

The young lady now walks without a perceptible limp.

The special points of interest that induce me to report the above case, are the number of fractures; the length of time between them; the fixed extension before applying the plaster dressing; and the complete immobility secured by running the plaster bandages over the pelvis to the waist; together with the perfect symmetry of the limbs, and the absence of deformity.

